# An Overview of Dietary Interventions and Strategies to Optimize the Management of Non-Alcoholic Fatty Liver Disease

**DOI:** 10.3390/diseases5040023

**Published:** 2017-10-22

**Authors:** Brandon J. Perumpail, Rosann Cholankeril, Eric R. Yoo, Donghee Kim, Aijaz Ahmed

**Affiliations:** 1 Department of Medicine, Drexel University College of Medicine, Philadelphia, PA 19129, USA; bjp63@drexel.edu; 2 Department of Medicine, Roger Williams Medical Center, Providence, RI 02908, USA; rtc1088@gmail.com; 3 Department of Medicine, Santa Clara Valley Medical Center, San Jose, CA 95128, USA; eric.r.yoo@gmail.com; 4 Division of Gastroenterology and Hepatology, Stanford University School of Medicine, Palo Alto, Stanford, CA 94305, USA; dhkimmd@stanford.edu

**Keywords:** nonalcoholic fatty liver disease—NAFLD, lifestyle intervention, weight loss, diet, exercise

## Abstract

**Aim:** To investigate the efficacy of lifestyle adjustment strategies as a preventive measure and/or treatment of obesity-related non-alcoholic fatty liver disease in adults. **Method:** A systematic review of literature through 1 July 2017 on the PubMed Database was performed. A comprehensive search was conducted using key terms, such as non-alcoholic fatty liver disease (NAFLD), combined with lifestyle intervention, diet, and exercise. All of the articles and studies obtained from the search were reviewed. Redundant literature was excluded. **Results:** Several types of dietary compositions and exercise techniques were identified. Most studies concluded and recommended reduction in the intake of saturated and trans fatty acids, carbohydrates, and animal-based protein, and increased intake of polyunsaturated fatty acids (PUFAs), monounsaturated fatty acids (MUFAs), plant-based proteins, antioxidants, and other nutrients was recommended. The Mediterranean and Paleo diet both seem to be promising schemes for NAFLD patients to follow. Exercise was also encouraged, but the type of exercise did not affect its efficacy as a NAFLD treatment when the duration is consistent. **Conclusions:** Although these different dietary strategies and exercise regimens can be adopted to treat NAFLD, current literature on the topic is limited in scope. Further research should be conducted to truly elucidate which lifestyle adjustments individually, and in combination, may facilitate patients with obesity-related NAFLD.

## 1. Introduction

Non-alcoholic fatty liver disease (NAFLD) is a significant global health concern that has been increasing at an alarming rate. NAFLD encompasses a wide spectrum of clinical disease from benign and non-progressive fatty infiltration of the liver to hepatic steatosis, accompanied by distinctive balloon degeneration (hallmark histologic feature), inflammation, and fibrosis (nonalcoholic steatohepatitis or NASH) in the absence of excessive alcohol consumption and other known causes of chronic liver disease [1]. NASH is associated with progressive liver damage leading to cirrhosis. Furthermore, simple steatosis has the potential to progress to fibro-cirrhotic disease. NASH-related cirrhosis can be complicated by end-stage liver disease (liver failure) and hepatocellular carcinoma (HCC), necessitating liver transplantation [1]. However, the occurrence of HCC in non-cirrhotic livers is being recognized at an increasing frequency [1]. Currently, NAFLD is the most common cause of chronic liver disease in the United States (US) adult population with a prevalence of 80–100 million and is the most rapidly rising indication for liver transplantation in the US [2,3]. Currently, NAFLD is considered the most common cause of chronic liver disease worldwide, with an estimated prevalence of 1 billion [2,3]. The global epidemic of obesity has increased the risk of metabolic syndrome [2,3]. In Western countries, NAFLD is associated with obesity and insulin resistance. However, NAFLD can develop at a lower BMI in Asians and without insulin resistance. In urban regions of Asia and some African countries, the globalization of western diet and lack of physical activity has led to a rise in the prevalence of NAFLD [2,3]. While rural areas in these continents have a much lower prevalence of NAFLD due to a high level of physical activity despite dependence on a predominantly carbohydrate-based diet [2,3]. The estimates of NAFLD prevalence in Asia range from 15–45% with 20% in China 27% in Hong Kong, and 15–45% (rural versus urban) in South Asia, South-East Asia, Korea, Japan, and Taiwan. The pandemic of obesity and diabetes have impacted Asia more recently in the setting of relatively lower degrees of adiposity than Europe and the US [2,3].

**Figure 1:**
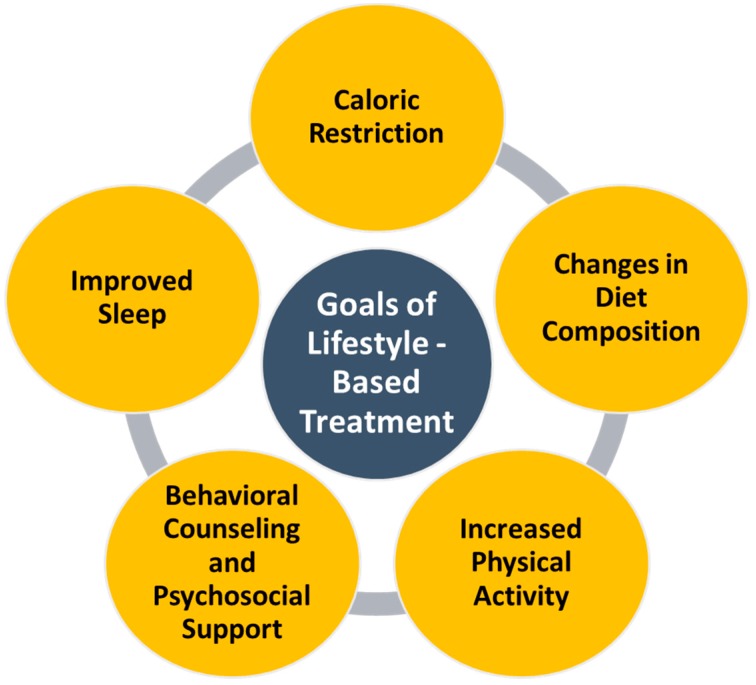
Goals of Lifestyle-Based Treatment.

The disease burden of NAFLD is significant with a trajectory to become the leading indication for liver transplantation in the next decade or so. Syndrome [4]. Therefore, stake holders and policy makers must make a concerted effort to prevent the development of NAFLD and its progression [4]. There are no approved pharmacological treatments for NAFLD in the US. However, direct and indirect data suggest that prevention of obesity with dietary modifications may reduce the risk of NAFLD and NASH-related complications. Weight loss is the primary treatment for obesity, a primary cause of NAFLD with common risk factors, such as cardiovascular disease, diabetes, hypertension, dyslipidemia, and metabolic syndrome [4]. NAFLD is also referred to as the hepatic manifestation of metabolic syndrome—an outdated and incomplete notion, which fails to render the bi-directional relationship of NAFLD with the metabolic syndrome. Status [2,5]. Cross-sectional studies have shown that features of the metabolic syndrome are strongly associated with the elementary histological features of NASH [2,5]. In addition, prospective data have shown that pre-existent NAFLD at baseline predicts the future development of both type II diabetes mellitus and metabolic syndrome over a short-time follow-up [2,5]. While weight loss alone in overweight and obese to ideal body weight may help to prevent and treat NAFLD, exercise, and other treatment interventions must be instituted on an individualized basis in lean NALFD patients who have underlying insulin resistance and metabolic irregularities [2,5]. A study showed that regardless of body mass index (BMI), weight loss of 2.7 ± 5.0 kg was significantly associated with NAFLD remission with 75% remission rate among NAFLD patients who lost 5% or more from their baseline weight [5]. Common attributes of NAFLD patients include many unhealthy habits, such as sedentary lifestyles and poor diet [6]. Patients with weight loss (at least 5% and ideally 10% weight loss) through lifestyle changes demonstrated resolution of NAFLD or improvement of fibrosis, further indicating that if individuals can alter their daily unhealthy habits sufficient weight loss can be achieved, and NAFLD counteracted [7]. The overall and basic goals of lifestyle-based treatment include caloric restriction, changes in diet composition, increased exercise, stress reduction, and improved sleep, and are indicated in Figure 1 [8].

The body fat mass is stabilized through balance between energy intake and energy expenditure, called energy homeostasis [8]. Weight loss can only occur when the energy input into the body is less than energy expenditure [8]. Diet and exercise are the most direct ways to accomplish this goal. However, when an external interference (such as, dietary modification or increased physical activity) changes the body fat mass, there is a counter-measure to restore the body to the previous fat mass set point [8]. After the interference ends, the body approaches fat mass noted before the intervention started [8]. Thus, lifestyle modification strategies should focus on maintenance programs with a goal to pursue sustained response, rather than a short-lived and transient benefit [9]. An individualized assessment should be employed to determine the proper lifestyle adaptation for each patient.

## 2. Diet Types

Improper dietary composition (high proportion of carbohydrates) or excessive caloric intake can lead to obesity and its comorbidities, including NAFLD [10]. Weight gain increases the risk of NAFLD and has been recognized as a reliable predictor of NAFLD [5]. Calorie restriction forms the backbone of effective NAFLD management. An interventional study found that weight loss was associated with reduction in hepatic steatosis [11]. The general guideline for caloric restriction is a decrease of 500–1000 calories from the daily requirement [4]. This suggests that men should have approximately 1200–1600 calories per day and women should aim to have approximately 1000–1200 calories per day, as shown in Table 1 [4]. However, caloric restriction alone may not always be beneficial in optimizing the management of NAFLD; the composition of the diet is a critical element and must be addressed.

### 2.1. Fat

**Figure 2:**
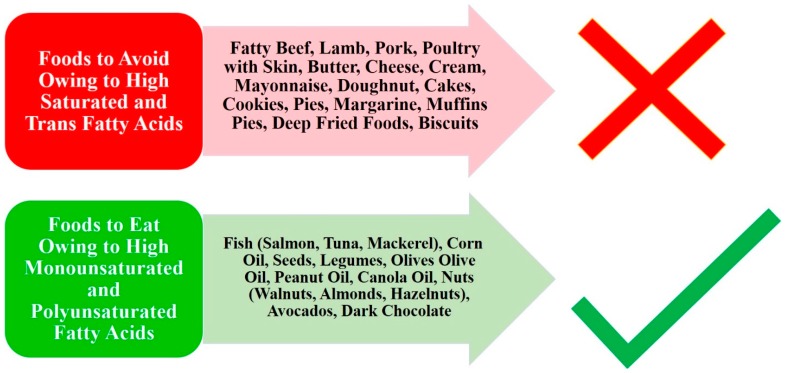
Examples of Dietary Fats.

Variation in fat molecular structure (type) causes them to have a range of effects on health [12]. Many NAFLD patients often have diets that include high saturated fatty acids, trans fatty acids, and cholesterol [13]. These types of fats come with many detrimental effects on insulin resistance, sugar homeostasis, cardiovascular disease, lipid metabolism, and NAFLD development or progression [9,14,15]. Mice studies with diets high in saturated fatty acids demonstrated an increased inflammation due to oxidative stress and extensive hepatocyte damage [4]. A study in humans showed that cholesterol was associated with hepatic inflammation and fibrosis [16]. On the contrary, low-fat diets are traditionally recommended and are effective in achieving sustainable weight loss [17]. The Acceptable Macronutrient Distribution Range (AMDR) for lipids is 20% to 35% of the daily diet in adults [18]. Trans and saturated fatty acids, as well as cholesterol, should be decreased to as low as possible and replaced with beneficial fat composition [19]. Polyunsaturated fatty acids (PUFAs), particularly Omega-3 fatty acid, docosahexaenoic acid (DHA), and eicosapentaenoic acid (EPA), have many protective effects when studied in patients with NAFLD [20]. They reduce hepatic steatosis through amplified fatty acid breakdown and downregulated lipid proliferation initiated by the peroxisome proliferator-activated receptor alpha [21]. Furthermore, omega-3 fatty acids interact with the inflammasome and G protein coupled receptor 120 (GPR120) and GPR40 in macrophages to decrease inflammation and promote insulin sensitization [22]. Monounsaturated fatty acids (MUFAs) have protective effects, such as improved fat distribution to adipose tissue rather than liver tissue, decreased detrimental lipids in the body (cholesterol, triglycerides, and VLDL), and increased HDL [4,12]. Examples of the various dietary fats are shown in Figure 2.

### 2.2. Protein

Compared to fats and carbohydrates, the clinical data studying the role of proteins in patients with NAFLD are limited [23,24]. Increased protein diets can support weight loss by increasing satiety, improving glucose homeostasis, increased meal-induced thermogenesis, protecting lean body mass, and increasing energy expenditure [23,24]. The substitution of carbohydrates with protein in studies indicated improved satiety and lower cholesterol and triglycerides levels with a decreased risk of cardiovascular disease in patients [25,26]. The recommendation for protein for patients with cirrhosis is up to 1.5 g/kg/day [4]. However, the source of protein remains an issue for debate [26]. Studies have shown that with proteins from animal sources and processed meat tend to also be high in fat and patients showed increased risk of diabetes and cardiovascular disease [26]. However, patients placed on soy-based protein showed decreased alanine transaminase (ALT) levels and hepatic steatosis, while protein from low-fat dairy, poultry, fish, and nuts promoted weight loss and reduced cardiovascular risk [12,24]. Thus, although there may be a beneficial impact of weight loss from protein diet, the effects on NAFLD from a protein-based diet are not fully understood.

### 2.3. Carbohydrates

A persistently higher amount and/or proportion of dietary carbohydrates in patients with NAFLD lead to increased insulin and triglyceride concentrations in the blood, lipogenesis, and decreased insulin sensitivity of the liver [27]. Both dietary fructose consumption and serum uric acid concentrations are independently associated with NASH and fructose consumption was independently linked with high serum UA concentrations [17]. A cross-sectional study found that increased dietary carbohydrates were associated with increased weight, BMI, blood pressure, insulin, and triglycerides [28]. Low-carbohydrate diets are more commonly being recommended for patients with NAFLD in the setting of obesity [23]. The AMDR for carbohydrates is between 45% and 65% of the daily diet in adults [18]. However, low-carbohydrate diets recommend less than 60 g per day and some even start with 20 g and slowly increase over time [23]. Low-carbohydrate diets have been associated with a reduction in circulating insulin, glucose, and triglycerides, while increasing HDL [17]. Randomized trials comparing low-fat diets and low-carbohydrate diets indicate that those with the low-carbohydrate diets achieved more weight loss in the same amount of time [23]. Another study that evaluated insulin resistance in obese patients given either a low-carbohydrate/high-fat diet (40% and 45%, respectively) versus a high-carbohydrate/low-fat diet (60% and 25%, respectively) demonstrated that the former group showed greatly reduced blood insulin and ALT, suggesting that low-carbohydrate diets may be more effective than a low-fat diet [12]. Furthermore, in a study comparing low-carbohydrate and high-carbohydrate hypocaloric diets in obese patients, the low-carbohydrate group had greater reduction in hepatic steatosis and glucose production; however, after a certain extent of weight loss the difference in hepatic steatosis was no longer significant [12]. Data are lacking and additional studies are needed to further understand and establish the duration of the effectiveness of low-carbohydrate diets.

### 2.4. Glycemic Index

More recently, glycemic index has gained attention as an important predictor of weight loss and progression of NAFLD [29]. The glycemic index is a measurement that compares dietary products based on how much is converted and absorbed into blood as glucose and is measured as a percentage [12]. High glycemic index foods include chocolate, cookies, pastries, and high starch food products. These items are common in the diets of a majority of patients with NAFLD and have been pathogenetically tied with insulin resistance, metabolic dysfunction, as well as increased hepatic fat [12,29]. When diet alteration included low glycemic index food products, patients showed decreased overall fat mass and hunger with increased lipid utilization and increased satiety [25,30,31]. Another study found that the lower glycemic index diet group showed lower hepatic fat and glycogen storage levels when compared to the other group with a higher glycemic index diet [29]. These recent findings may suggest that diets with lower glycemic loads may benefit patients with NAFLD.

### 2.5. Antioxidants

A characteristic of the progression of NAFLD to NASH is the oxidative stress seen that leads to hepatic inflammation, fibrosis, and cirrhosis [32,33,34,35]. Dietary supplements and herbs are marketed worldwide and are consumed with regular diet despite unproven efficacy and reports of hepatotoxicity [32,33,34,35]. These products may contain several ingredients (Herbalife™ products, Hydroxycut™, LipoKinetix™, UCP-1, and OxyELITE™), while others have a single ingredient (green tea extract, linoleic acid, usnic acid, 1,3-Dimethylamylamine, vitamin A, Garcinia cambogia, and ma huang) [32,33,34,35]. We recommend caution with products lacking prospective data and robust testing. Dietary antioxidants can become a powerful tool for reducing this cellular stress and preventing NAFLD progression [36]. Vitamin E is an antioxidant that has been studied in NAFLD and has been found to decrease aspartate transaminase (AST) and ALT levels, inflammation, hepatocellular ballooning, and steatosis when included in the diet [35]. Vitamin D deficiency is often noted in NAFLD patients and has been associated with hepatic inflammation and metabolic syndrome [36]. Vitamin D supplementation showed improvement in NAFLD by improving insulin secretion and insulin responsiveness, promoting anti-inflammatory factors, and down-regulating pro-fibrotic factors [36]. Vitamin C has also been tied to improved liver histology. Resveratrol, present in red wine and grapes, promotes weight loss, decreases oxidative stress, promotes insulin sensitivity, and decreases inflammation, blood pressure, liver fat, blood glucose, and triglycerides [12]. Green tea is rich with antioxidant potential in that it has polyphenolic catechins that reduce inflammation, promote thermogenesis, and decreased overall lipids [27]. Research indicates that increasing antioxidant-rich food with diet or taking supplements have favorable NAFLD-related outcomes.

### 2.6. Coffee

Recent literature has shown a link between coffee drinking and NAFLD regression. Prospective studies have shown increased coffee drinking associated with significantly lower risk for hepatic fibrosis predicting that coffee consumption may have protective effects on NAFLD [37]. Although it is currently unknown which ingredients in coffee make it beneficial, scientists believe the caffeine or polyphenols are players in the effectiveness of coffee in preventing hepatic damage [13].

### 2.7. Bile Acids, Choline, and Fiber

Bile acids have an important role in lipid absorption, but also control energy balances by modulating energy expenditure, meal-induced thermogenesis, and regulating satiety and hunger [38]. Thus, bile acids could be effective in preventing weight gain and even promote weight loss [38,39]. Mice fed diets with cholic acid conjugates experienced decreased triglycerides, liver steatosis, plasma fatty acids, and lipogenesis [39]. Thus, adding food that stimulates bile acid formation to daily diet patterns could be beneficial in patients with NAFLD.

Choline is a nutrient that can be included in the diet or produced in the body and is metabolized in the liver to produce betaine [40]. However, endogenous choline sources do produce enough to meet the body requirements, leaving an individual in a state of choline deficiency [40]. This increases de novo hepatic lipogenesis, decreases bile acid synthesis, and promotes cholesterol accumulation, which leads to hepatic steatosis that eventually progresses to NAFLD [40]. Current literature is uncertain whether the beneficial effects of choline are dependent on other co-factors or not [40].

Dietary fiber has been connected to many beneficial effects on metabolism and NAFLD [12]. A comparative study found that fiber is low in many NAFLD diets and that increased consumption of foods with fiber reduces insulin resistance, improves hepatic steatosis, and lowers LDL levels [41]. Other analyses have shown that fiber increase satiety, reduced the absorption rate of carbohydrates, and increased incretin secretion to promote insulin secretion [12].

### 2.8. Probiotics

Recent data have implicated gut-liver axis in the pathogenesis of NAFLD and supported the role of microbiota as a casual factor [27,42,43,44]. Probiotic replenish commensal bacteria with the ability to modulate the microbiota and impact health. Recent data have suggested a range of potential health benefits from probiotics, including the protective effects of Lactobacillus kefiri in preventing high-fat-diet-induced obesity by direct reduction of cholesterol [27,42,43,44]. High fat and high carbohydrate diet stimulates the pathogenesis of NAFLD [27,42,43,44]. Gut microbiota-mediated regulation of lipid profiles was dependent on dietary lipids and carbohydrates [27,42,43,44]. Probiotics retard the progression of high fat and sucrose diet-induced steatosis through its effects on leptin, resistin, and inflammatory biomarkers [27,42,43,44]. Changes in the microbiota of the gut can alter absorption and energy storage by the gut-liver axis though the activation of Toll-like receptors (TLR), which promote an inflammatory response [42,43]. Normally the liver has a high resistance to the TLR ligands produced by the gut microbiota, but changes in gut composition lead to the deterioration of this liver tolerance and increased inflammation seen in NAFLD patients and progression to NASH [42,43,44]. Current studies suggest that the renormalization of the gut microbiota with probiotics or prebiotics is a promising method of NAFLD management; however, more research on this topic must be complete to better understand the role of probiotics [27,42,43,44].

### 2.9. Mediterranean and Other Diets

There are many diets that have gained increased interest for weight loss, as summarized in Table 2. The Mediterranean diet, modeled after meals in the Mediterranean areas, has been adopted by many in hopes of weight loss [45]. The constituents of the Mediterranean diet including olive oil, fish, nuts, whole grains, fruits, and vegetables have been shown to negatively correlate with NAFLD, while components of Western diet such as soft drinks, fructose, meat, and saturated fatty acids have been shown to promote the pathogenesis of NAFLD [45,46]. The Mediterranean diet embodies a healthy diet that aids in the prevention of metabolic syndrome, cardiovascular disease, obesity, and NAFLD [45,46]. This diet is characterized as low-carbohydrate (40% of calories) and in contrast to low-fat diets, the Mediterranean diet includes fat as 40% of the diet, emphasizing the intake of MUFAs, olive oil, nuts, vegetables, fruits, fish, poultry, and legumes [31,45,46,47]. The diet replaces saturated and trans fatty acids with MUFAs and PUFAs [19]. The Mediterranean diet decreases the intake of processed meat, red meat, and leans towards a more plant-based diet [19,23,31,45,46,47]. The diet also promotes drinking red wine (source of resveratrol) in moderation [47]. With all of these healthy choices in the diet, the Mediterranean is a great diet route for patients with NAFLD or even those looking for preventative measures [31,45]. An observational study with this dietary intervention for six months as NAFLD treatment demonstrated that the participants with the diet intervention showed significant improvement in BMI, waist circumference, waist-to-hip ratio, cholesterol, ALT, AST, triglycerides, blood glucose, hepatic steatosis, and significant overall reduction in NAFLD severity [45]. Another study showed weight loss and improved liver function in NAFLD patients who adhered to the Mediterranean diet [19,46,47]. These findings strengthen the Mediterranean diet as a valid approach to treat NAFLD.

Another common diet that does not have much literature in NAFLD is the Hunter-Gather or Paleo diet. This diet is modeled after diets of people who lived as hunter-gathers during the Paleolithic Era [48,49]. The Paleo diet is moderate-fat with fats accounting for approximately a third of the calories [49]. Saturated and trans fatty acids are replaced by MUFAs and PUFAs from game and marine animals rather beef, chicken, pork, and processed meats [49]. Carbohydrates are also reduced to a moderate amount (45–65%) with reduced grains and most carbohydrates coming from fruits and vegetables unlike the Mediterranean diet that includes more grains and sugar; thus, making the Paleo more effective at improving insulin resistance and cardiovascular disease [47,49]. Randomized trials have found that those with the Paleo diet intervention had decreased markers of metabolic syndrome such as decreased blood pressure, weight, cholesterol, and triglycerides, with increased HDL [48]. These indications may suggest that the Paleo diet may be an effective approach for NAFLD, but more research is needed to directly connect this diet scheme with improvement in NAFLD.

## 3. Other Lifestyle Changes

Apart from diet composition, meal timing is an important factor that can be taken into consideration. Calories consumed in three meal sessions are more conducive to weight loss and liver health [50]. A randomized study found that when compared against a group who had three meals with the same total daily calories, the group with the three smaller meals plus snacks in between showed increased triglycerides, overall fat, weight gain, and hepatic steatosis [50].

Furthermore, patients with NAFLD are more likely to have a sedentary behavior, which is a risk factor for NAFLD [46]. A lower level of physical activity is associated with increased liver and all-cause mortality [51]. Extended durations of sedentary activity was tied with NAFLD and a cross-sectional study found that adding breaks in sedentary time lead to decreased weight, triglycerides, and blood glucose [51,52,53]. Cross-sectional studies found that sedentary behaviors were linked with unhealthy dietary patterns; participants who reported sedentary lifestyles through increased television viewing and recreational internet use for more than 4 h a day also reported increased snacking and inverse association with plant-based diets or health consciousness [6].

Exercise is a means of increasing energy expenditure versus energy intake to facilitate weight loss. The three main exercise techniques are aerobic exercise, resistance exercise, and high intensity intermittent exercise; however, intensity of workout measured by oxygen consumption does not change effectiveness as NAFLD treatment [46]. A meta-analysis including twenty randomized control trials with 1073 NAFLD patients concluded that exercise alone or combined with dietary intervention is beneficial with an improvement in liver enzymes and hepatic histology [54]. Furthermore, exercise is noted to have a beneficial effect on intrahepatic triglycerides, even in the absence of weight loss [54]. A study comparing aerobic exercise versus resistance exercise, the latter was able to have the same liver fat decrease with less energy consumption and intensity, making it more easily adoptable by NAFLD patients [54]. The recommendation for physical activity duration and frequency was 40–45 min sessions that occur three times a week, [51,54]. Studies showed that exercise improved insulin sensitivity, lipid circulation, energy balance, but exercise is more effective when paired with diet [23,46,51,54]. The effects of exercise on NAFLD patients are still not fully understood and further research on this topic would help elucidate how exercise can act as treatment for NAFLD.

## 4. Conclusions

There are many types of diet compositions and exercise techniques that can be adopted to modify the lifestyles of patients with NAFLD in an effort prevent, treat, and or retard the progression of disease. Current evidence-based data demonstrates that reduced intake of saturated and trans fatty acids, carbohydrates, and animal-based protein with increased intake of PUFAs, MUFAs, plant-based protein, antioxidants, and other nutrients should be recommended and is beneficial. The Mediterranean and Paleo diet both seem to be new and promising schemes for patients with NAFLD. Exercise was also encouraged, but the intensity of workout did not change effectiveness. Although these different diet strategies and exercise forms can be adopted to treat NAFLD, current literature on this topic is limited in scope. Further research should be pursued to truly elucidate the best lifestyle adjustments to optimize the management of NAFLD.

## Figures and Tables

**Table 1:** Recommendations for Macronutrients.

Macronutrient Recommendations	
Daily Calories	1200–1600 calories (Men), 1200–1200 calories (Women)
Lipids	20–35% of daily calories with increased MUFAs and PUFAs
Proteins	1.5 g/kg/day with emphasis on plant-based protein
Carbohydrates	≤45–65% of daily calories with decreased simple sugars

Abbreviations: MUFA: Monounsaturated Fatty Acid; PUFA: Polyunsaturated Fatty Acid.

**Table 2:** Comparison of Mediterranean and Paleo Diets.

Mediterranean Diet	Paleo Diet	
Lipids	Moderate Fat 40% of daily calories	Moderate-Low Fat 35% of daily calories
Proteins	Reduce processed and red meat. Increased plant-based protein, fish and poultry	Reduce processed and red meat. Increased plant-based protein, and meat from marine and game animals
Carbohydrates	Low Carbohydrate ≤40% of daily calories Fruits, vegetables, sugar, and grains	Moderate Carbohydrates ≤45–65% of daily calories Fruits and vegetables Decreased grains
